# Protocatechuic aldehyde acts synergistically with dacarbazine to augment DNA double-strand breaks and promote apoptosis in cutaneous melanoma cells

**DOI:** 10.1186/s12906-023-03933-w

**Published:** 2023-04-06

**Authors:** Junxia Pei, Zhou Su, Xin Zeng, Ya Zhong, Yamei Zhang, Yixi Yang, Qiuxia Lu, Jian Li, Yu Deng

**Affiliations:** 1 Engineering Research Center of Sichuan-Tibet Traditional Medicinal Plant, Chengdu University, Chengdu, 610106 China; 2 Institute of Cancer Biology and Drug Discovery, Chengdu University, Chengdu, 610106 China; 3 School of Food and Biological Engineering, Chengdu University, Chengdu, 610106 China; 4 School of Pharmacy, Chengdu University, Chengdu, 610106 China; 5 Key Laboratory of Clinical Genetics, Affiliated hospital of Chengdu University, Chengdu, 610106 China; 6 School of Basic Medical Sciences, Chengdu University, Chengdu, 610106 China

**Keywords:** Melanoma, Protocatechuic aldehyde, Dacarbazine, Synergy, MGMT

## Abstract

**Background:**

Despite rapid developments in immunotherapy and targeted therapy, dacarbazine (DTIC)-based chemotherapy still has been placed at the first-line for advanced melanoma patients who are after failure of immunotherapy or targeted therapy. However, the limited response rate and survival benefit challenge the DTIC-based chemotherapy for advanced melanoma patients.

**Methods:**

Two melanoma cell lines, A375 and SK-MEL-28 were cultured with PA and DTIC over a range of concentrations for 72 h and the cell viabilities were detected by CCK8 assay. The Bliss model and ZIP model were used for calculating the synergistic effect of PA and DTIC. DNA double-strand breaks in the two cell lines were examined by the Comet assay, and cell apoptosis was analyzed by flow cytometry. The short hairpin RNA (shRNA)-mediated knockdown, Real-time polymerase chain reaction (RT-PCR) and Western blot were performed for molecular analysis.

**Results:**

In the present study, we report that Protocatechuic aldehyde (PA) synergistically enhances the cytotoxicity of DTIC to two melanoma cell lines, A375 and SK-MEL-28. The combination of PA and DTIC augments DNA double-strand breaks and increases cell apoptosis. Further mechanism study reveals that PA destabilizes MGMT protein (O-6-Methylguanine-DNA Methyltransferase) through the ubiquitin-proteasome process and directly repairs DTIC-induced genetic lesions. Knockdown of MGMT compromises the synergistic effect between PA and DTIC.

**Conclusion:**

Our study demonstrates that the bioactive compound, Protocatechuic aldehyde, synergistically promotes the cytotoxicity of DTIC to melanoma cells through destabilization of MGMT protein. It could be a potential candidate for melanoma chemotherapy.

**Supplementary Information:**

The online version contains supplementary material available at 10.1186/s12906-023-03933-w.

## Background

Cutaneous melanoma is the most aggressive and lethal skin cancer. Although there has been huge progression in immune checkpoint inhibitors (ICI) and BRAF or MEK targeted therapy, dacarbazine (DTIC)-based chemotherapy still stands at the first-line for advanced melanoma patients who are after failure of immunotherapy or targeted therapy [1, 2]. The major challenge for DTIC-based chemotherapy is low response rate (about 10-15%) and no significant survival benefit even in combination with other chemotherapy drugs [3, 4]. Therefore, promotion of DTIC efficacy to fight advanced melanoma is urgently required.

DTIC or its analogue temozolomide (TMZ) is an alkylating agent that refers alkyl groups to O6 position of guanine forming O6-methylguanine (O6-MeG). DNA containing O6-MeG is prone to be mismatched with T at the opposite strand. Regardless of whether the O6-MeG is matched with T or C, the DNA mismatch repair system recognizes them as mismatched nucleotides, and proceeds to excise and degrade the strand opposite to the O6-MeG. The continuous excision process will generate DNA double-strand breaks and trigger cell apoptosis [5]. MGMT (O-6-Methylguanine-DNA Methyltransferase) is a unique gene in human cells that directly repairs O6-MeG DNA damages by removing the alkyl groups from guanine. High expression levels of MGMT in advanced melanoma patients correlate with resistance to DTIC [6, 7] and poor survival [8]. Likewise, modulation of MGMT expression or activity is expected to be a promising therapeutic target to promote the efficacy of DTIC/TMZ in cancers [9–14].

Protocatechuic aldehyde (PA) is a bioactive compound isolated from a traditional Chinese herb, *Salvia miltiorrhiza* [15]. Several studies demonstrated that PA possessed activities of antioxidation and anti-inflammation [15–18]. We previously reported that PA transcriptionally controlled expression of p21 and E-cadherin via direct binding to the dehydrogenase domain of C-terminal binding protein 1(CtBP1), a transcriptional co-repressor, to inhibit growth and migration of breast cancer cells [19]. In this study, we found that PA reduced MGMT protein levels in melanoma cells via destabilization of MGMT rather than transcriptional regulation, and acted synergistically with DTIC to augment DNA damages and apoptosis in melanoma cells.

## Materials and methods

### Cell culture and reagents

Human cutaneous melanoma cell lines A375 and SK-MEL-28 were obtained from the Cell Bank of the Chinese Academy of Sciences. Cells were maintained in high glucose DMEM medium (Wisent, St-Bruno, Canada) supplemented with 10% fetal bovine serum (Wisent, St-Bruno, QC, Canada) at 37◦C with 5% CO_2_.

Protocatechuic aldehyde (PA, D108405, Sigma, Shanghai, China) and Dacarbazine (DTIC, S122104, Selleck, Shanghai, China) were dissolved in dimethyl sulfoxide (DMSO) as stock solutions at the concentrations of 200mM and 50mM. Cycloheximide (CHX, 66-81-9, Selleck, Shanghai, China) was dissolved in PBS at the concentration of 15 mg/ml. MG132 (S2619, Selleck, Shanghai, China) was dissolved in DMSO as a stock solution at the concentration of 50mM.

### Cell viability and synergistic effect analysis

A375 and SK-MEL-28 cells were planted in 96-well plates at a density of 2000 cells per well. After the 72 h treatments with PA, DTIC or their combination. Cell viability was detected by the Cell Counting Kit-8 assay (CCK8) (C0039, Beyotime Biotechnology, Shanghai, China).

SynergyFinder 3.0 software (https://synergyfinder.fimm.fi) was used for evaluating synergistic effects and calculating synergy scores following the online instructions.

### Western blot analysis

Cells were lysed by RIPA150 lysis buffer as previously described [19]. Protein samples were loaded for sodium dodecyl sulfate-polyacrylamide gel electrophoresis (SDS-PAGE) and transferred to polyvinylidene fluoride (PVDF) membranes (Bio-Rad, Hercules, CA, USA). Membranes with protein were blocked with 5%(w/v) skim milk at room temperature for 1 h. Then incubated with primary antibodies at 4℃ for overnight, followed by 1 h incubation with Horseradish Peroxidase (HRP) -conjugated secondary antibodies at room temperature. After 3 times washing with 1 × TBST buffer, SuperLumia ECL HRP Substrate Kit (K22020, Abbkine, Shanghai, China) was used for signal detection.

Primary antibodies are listed as below: GAPDH(60004-1-Ig, Proteintech, Wuhan, China), MGMT(ER40104, HUABIO, Hangzhou, China), γ-H2AX(Ser139)(ET1602-2, HUABIO, Hangzhou, China), caspase-3(9662, Cell Signaling Technology, Danvers, MA, USA), cleaved caspase-3(ab32042, Abcam, Cambridge, UK).

### Real-time polymerase chain reaction (RT-PCR)

The RNA isolation and Real-Time PCR assay were described previously [20]. 18s expression was used as an internal control for normalization. Relative gene expression was calculated by the 2-∆∆Ct method. All samples were repeated at least three times.

The sequences of primers used for Real-Time PCR are as follows.

MGMT Forward: GCTGAATGCCTATTTCCACC

MGMT Reverse: ACTTCTCCGAATTTCACAACC

18 S Forward: TGACGGAAGGGCACCACCAG

18 S Reverse: GCACCACCACCCACGGAATC

### Comet assay

Neutral comet assay was performed by the comet assay kit (4250-050-K, R&D systems, Minnesota, USA) following the manufacturer’s manual [21]. Slides were stained with SYBR Gold and images were taken by the Nikon Eclipse Ni–U microscope (Nikon, Tokyo, Japan). For data quantification, five visual fields with at least 20 cells were randomly selected for analysis. Tail Moment was defined as Tail DNA% × Tail Length and was quantified using the Comet Assay Software Project (CASP).

### Flow cytometry analysis of cell apoptosis

A375 and SK-MEL-28 cells were stained with Annexin V-FITC and propidium iodide (PI) provided by the apoptosis-detection kit (AD10, DOJINDO, Kyushu, Japan). Cell apoptosis was detected by BD FACSAriaIII (B607, New York, USA) and the data were analyzed by FlowJo V10.

### Short hairpin RNA (shRNA)-mediated MGMT knockdown

Two different MGMT-targeted shRNAs and scramble oligos were cloned into PLKO.1-neo vector, respectively. (Plasmid#13,425; Addgene, Cambridge, USA) following the Addegen online protocol at https://www.addgene.org/protocols/plko/. The constructs containing shRNAs were confirmed by Sanger sequencing and were transfected into 293T cells by the Lipofectamine 2000 (Invitrogen, Carlsbad, CA) with the packing plasmids psPAX2 and pMD2 to produce lentivirus.

A375 and SK-MEL-28 cells were co-cultured with the lentivirus for 24 h and then selected with 1 mg/mL G418 for 10 days. The knockdown efficiency was determined by Western blotting. The shRNA oligos used in the study are listed below:

shMGMT-1: GCTGCTGAAGGTTGTGAAATT

shMGMT-2: GGAGGAGCAATGAGAGGCAAT

Scramble: CCTAAGGTTAAGTCGCCCTCG

### Statistical analysis

All experiments were performed at least three times. Statistical comparisons were conducted by the GraphPad Prism 8.0 software and analyzed by Student t-test or multiple comparisons were performed by one-way ANOVA with Tukey’s post-hoc tests. *P* < 0.05 was considered statistically significant.

## Results

### PA synergizes with DTIC in killing melanoma cells

**Fig. 1:**
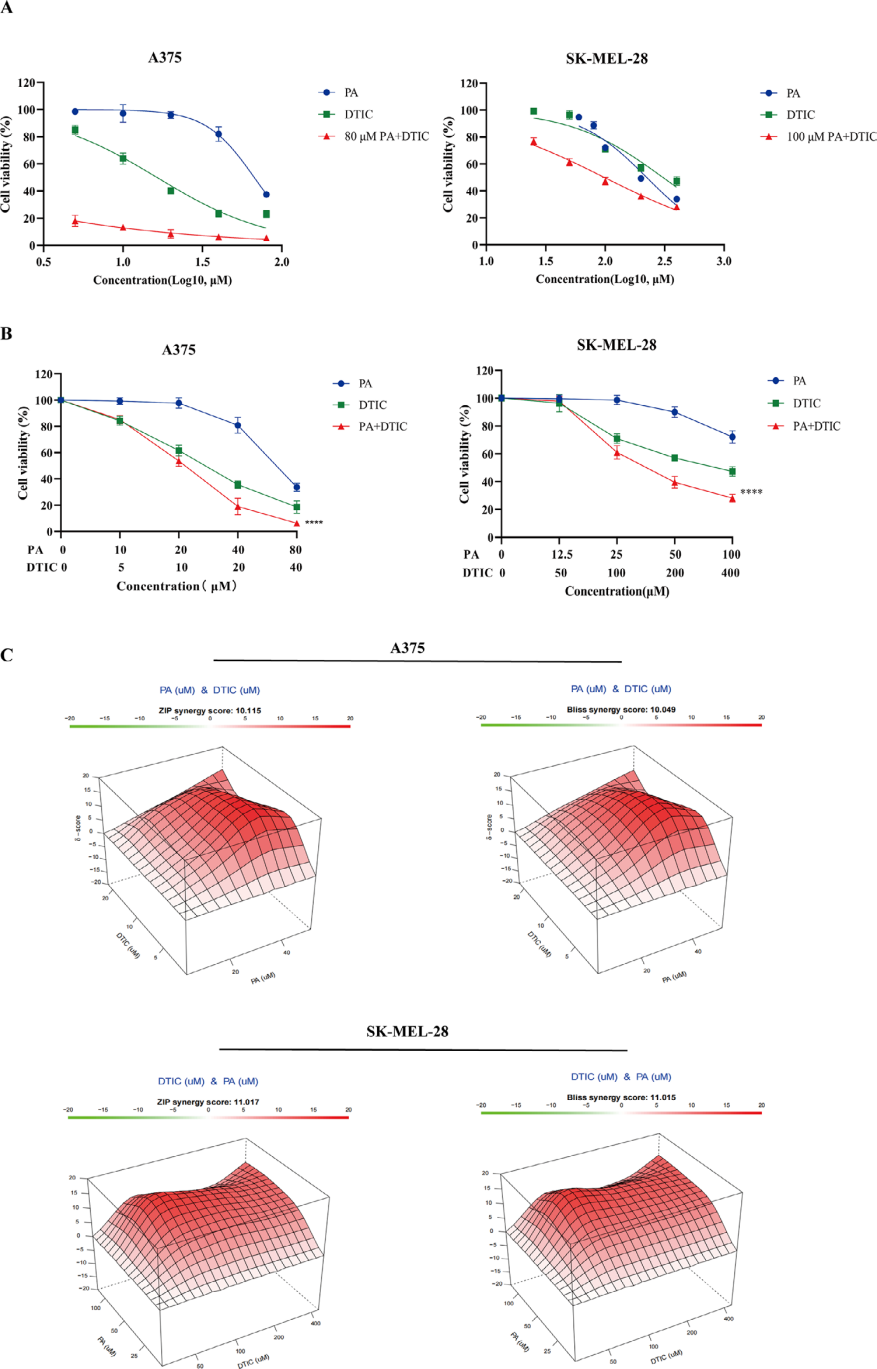
Protocatechuic aldehyde synergistically enhances DTIC cytotoxicity to melanoma cells. **A** Dose-response curves for PA, DTIC or DTIC combined with a certain concentration of PA for 72 h in A375 and SK-MEL-28 cells. **B** Dose-response curves for A375 and SK-MEL-28 cells treated with a range of concentrations of PA, DTIC or their combinations. **C** Synergy scores were calculated from the data represented in **B** for PA combined with DTIC for A375 and SK-MEL-28 cells. The left panel represents the synergy scores from ZIP model. The right panel represents the synergy scores from Bliss model. **** *p*<0.0001

Initially, we assessed the cytotoxicity of DTIC to two melanoma cell lines, A375 and SK-MEL-28, in the presence or absence of PA at the concentration of 80µM and 100µM, respectively. The 72 h IC_50_ of DTIC for A375 and SK-MEL-28 were 15.40 ± 1.39 µM and 309.55 ± 5.73 µM. By contrast, the IC_50_ of DTIC for the 2 cell lines were 0.28 ± 0.07 µМ and 98.20 ± 0.37 µM in the presence of PA (Table 1; Fig. 1A). The results indicated that PA may synergistically enhance the cytotoxicity of DTIC to melanoma cells.

**Table 1:** PA increases melanoma cells’ drug sensitivities to DTIC. A375 and SK-MEL-28 cells were treated with a range of concentrations of DTIC with or without PA for 72 h. Cell viability and IC_50_ were detected by CCK8 assay

	IC_50_(µM,mean ± SD)		*p* value	IC_50_(µM,mean ± SD)		*p* value
	**A375**	**A375 + 80µM PA**		**SK-MEL-28**	**SK-MEL-28 + 100µM PA**	
DTIC	15.40 ± 1.39	0.28 ± 0.07	*P <* 0.01	309.55 ± 5.73	98.20 ± 0.37	*P <* 0.01

Next, to determine whether PA potentiated the cytotoxicity of DTIC, A375 and SK-MEL-28 cells received a combined treatment of DTIC and PA over a range of concentrations for 72 h. Notably, PA potentiated the cytotoxicity of DTIC in the two melanoma cell lines, and exhibited a synergistic effect, which was determined by the ZIP model and the Bliss model (a synergy score > 10 is considered as synergistical) (Fig. 1B-C). [22]

### PA synergizes with DTIC in augmenting DNA double-strand breaks and promoting apoptosis

**Fig. 2:**
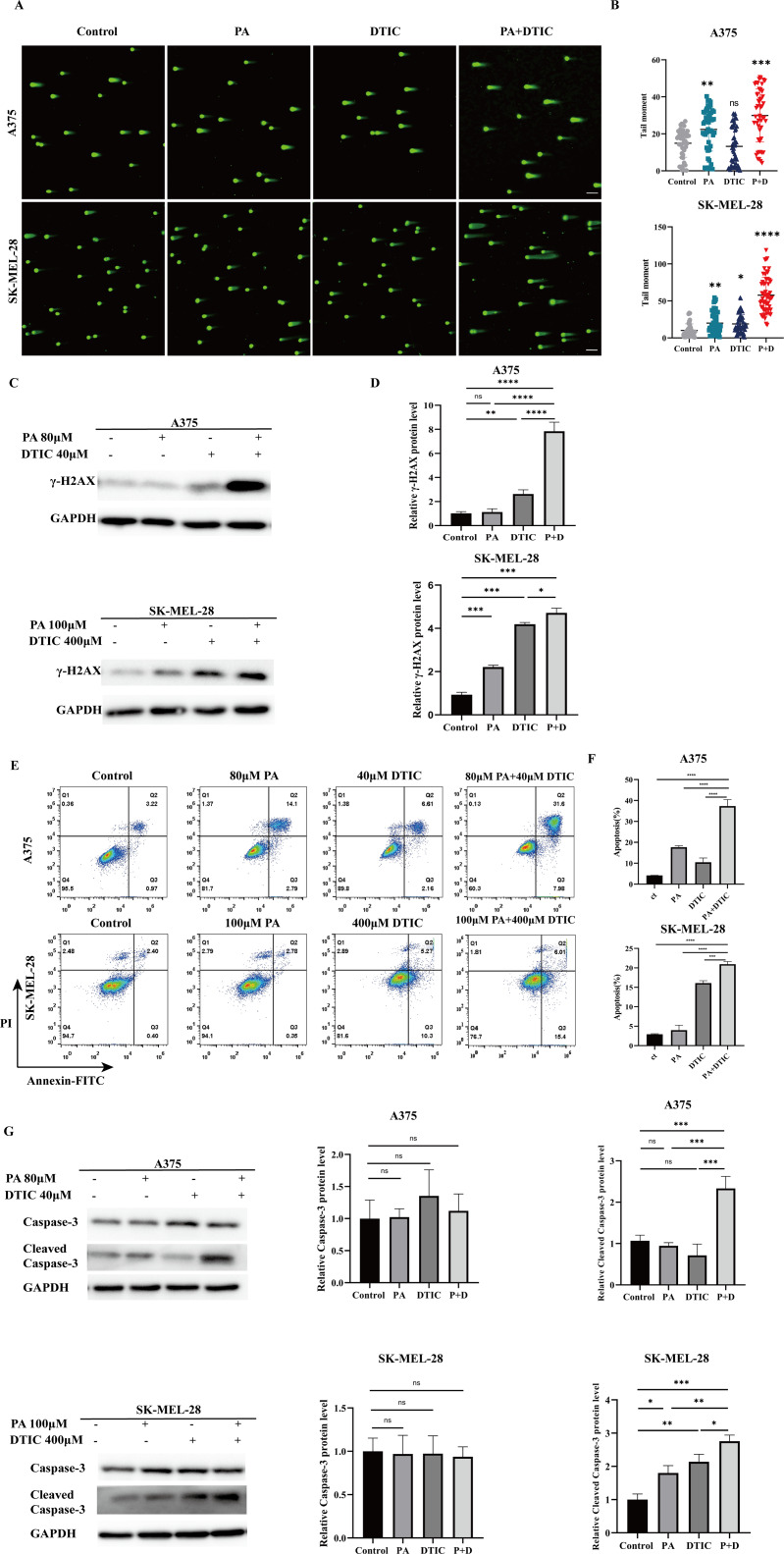
Combination of PA and DTIC increased DNA-double strand breaks and apoptosis in A375 and SK-MEL-28 cells. **A** The double-strand breaks in A375 and SK-MEL-28 cells were assessed by neutral comet assay. **B** Quantification of DNA percentages in comet tails in **A**. **C** γ-H2AX levels after 72 h of treatment with DMSO, PA, DTIC or combination of PA and DTIC in A375 and SK-MEL-28 cells. **D** Quantification of the relative γ-H2AX levels in **C**. **E** Cell apoptosis was analyzed by flow cytometry. The combined treatment showed the most apoptosis induction. **F** Quantification of apoptosis ratios in **E**. **G** Cleaved caspase-3 protein levels after 72 h of treatment with DMSO, PA, DTIC or combination of PA and DTIC in A375 and SK-MEL-28 cells. Right panel represents quantification of the relative cleaved caspase-3 protein levels in **G**. ns = no significant,*p < 0.05, **p < 0.01,***p < 0.001 and **** *p*<0.0001

DTIC facilitates O6-MeG formation to generate DNA double-strand breaks, thus inducing cell apoptosis. To evaluate whether PA could synergize with DTIC to increase DNA double-strand breaks, we carried out the comet assay to assess DNA double-strand breaks in A375 and SK-MEL-28 cells which were treated with DTIC or PA alone, or combination of DTIC and PA. In contrast to the marginal increase in double-strand breaks induced by DTIC alone, the single PA treatment induced a more clear increase in double-strand breaks. However, the combined treatment boosted the double-strand breaks in A375 and SK-MEL-28 as compared to the single agent application (Fig. 2A-B). Moreover, the sensitive biomarker of DNA double-strand breaks, γ-H2AX, was highly elevated in the combined treatment group (Fig. 2C-D).

**Fig. 3:**
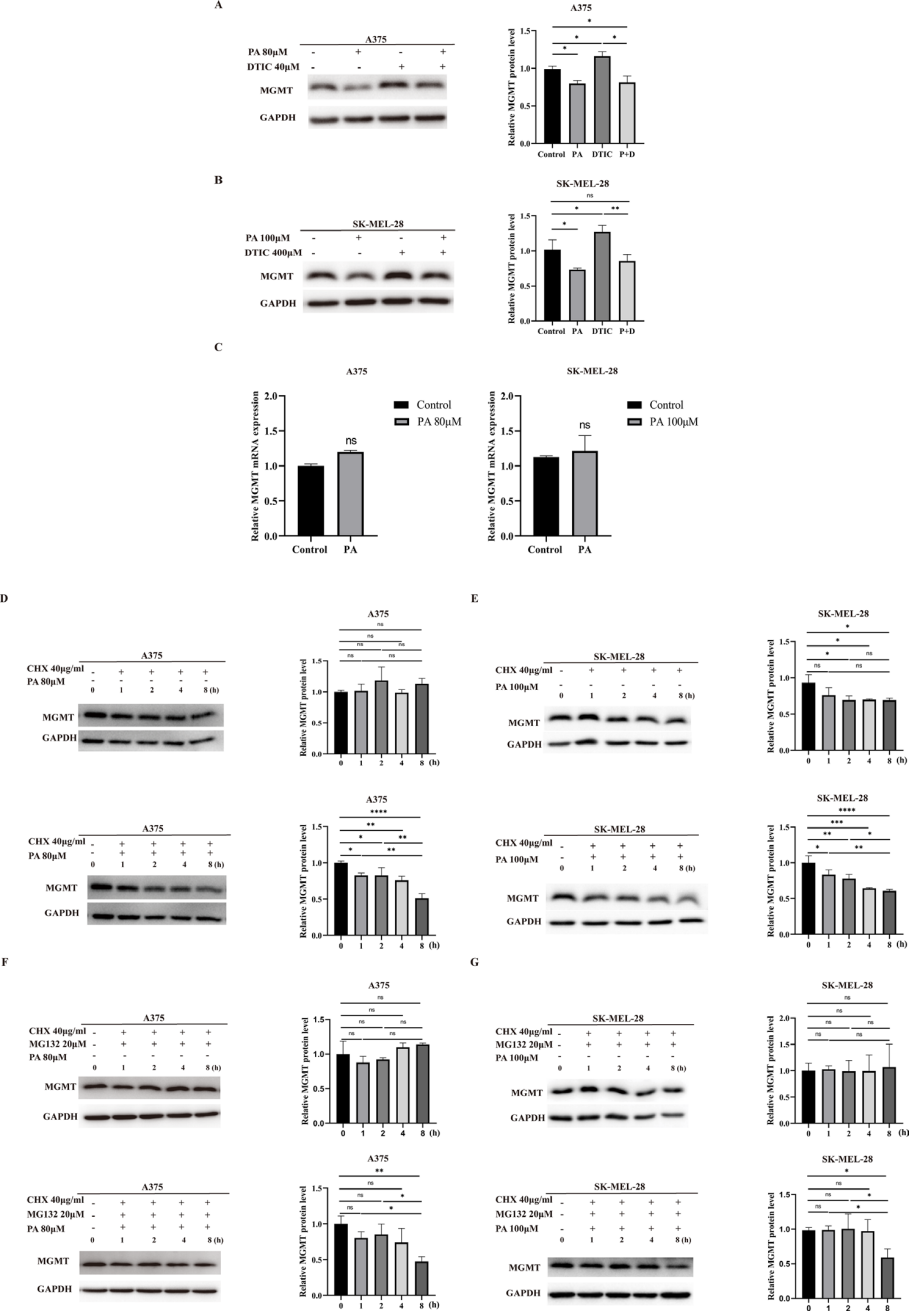
PA promotes MGMT degradation in melanoma cells. **A-B** Left panel shows western blot analysis for MGMT levels in A375 and SK-MEL-28 cells after 72h of treatment with DMSO, PA, DTIC or combination of PA and DTIC. Right panel represents the quantification of the MGMT protein levels for the left panel. **C** MGMT mRNA expression in A375 and SK-MEL-28 after 72 h PA treatment. **D-E** A375 and SK-MEL-28 were treated with cycloheximide in the presence or absence PA for 0-8 h hours. Western blot showed the MGMT degradation rates. Right panel represents quantification of the MGMT protein levels. **F-G** A375 and SK-MEL-28 were treated with MG132 combined with cycloheximide in the presence or absence of PA for 0-8 h hours. The MGMT protein levels were analyzed by western blot. Right panel represents quantification of the MGMT protein levels. ns = no significant, *p < 0.05, **p < 0.01,***p < 0.001 and **** *p*<0.0001

Since accumulated DNA double-strand breaks is a major cause of cell apoptosis, we analyzed cell apoptosis by flow cytometry. Similar with the DNA double-strand breaks formation, combination of DTIC and PA induced the most highly apoptosis and cleaved caspase-3 levels in the two melanoma cell lines (Fig. 2E-G).

### PA destabilizes MGMT in melanoma cells

MGMT directly repairs the DTIC-induced genetic lesions and confers DTIC/TMZ resistance properties to cancer cells. Therefore, we examined the MGMT expression in A375 and SK-MEL-28 after 72 h treatment with DTIC, PA, or their combination. The results showed that the two melanoma cell lines elevated their MGMT levels in response to DTIC treatment. But PA remarkably reduced the MGMT protein levels either treated alone or in combination with DTIC, indicating that PA may regulate MGMT expression in the two cell lines (Fig. 3A-B).

To address how PA regulated MGMT expression in melanoma cells, we first assayed MGMT mRNA expression in the two melanoma cell lines after receiving PA treatment for 72 h and found no significant changes at the mRNA level (Fig. 3C), suggesting that PA did not transcriptionally regulate MGMT. Next, we treated A375 and SK-MEL-28 with cycloheximide to block de novo protein synthesis in the presence or absence of PA and used western blotting to analyze MGMT protein levels in the two cell lines. The MGMT levels declined more slowly in the cells in the absence of PA by comparison with those cells with PA treatment (Fig. 3D-E). Ubiquitination of MGMT is a major biological process for its degradation. To further understand whether PA destabilizes MGMT through the ubiquitin-proteasome way, we used 20µM MG132 to block proteasome-mediated protein degradation in cycloheximide treated cells, with or without PA. The results showed that MG132 not only slowed down intrinsic MGMT degradation in single cycloheximide treated cells but also attenuated PA-mediated MGMT degradation in the melanoma cells (Fig. 3F-G). These data demonstrated that PA destabilized MGMT and promoted its degradation in A375 and SK-MEL-28 cells through proteasome-mediated protein degradation.

### Depletion of exogenous MGMT attenuated the synergy effect of PA and DTIC

**Fig. 4:**
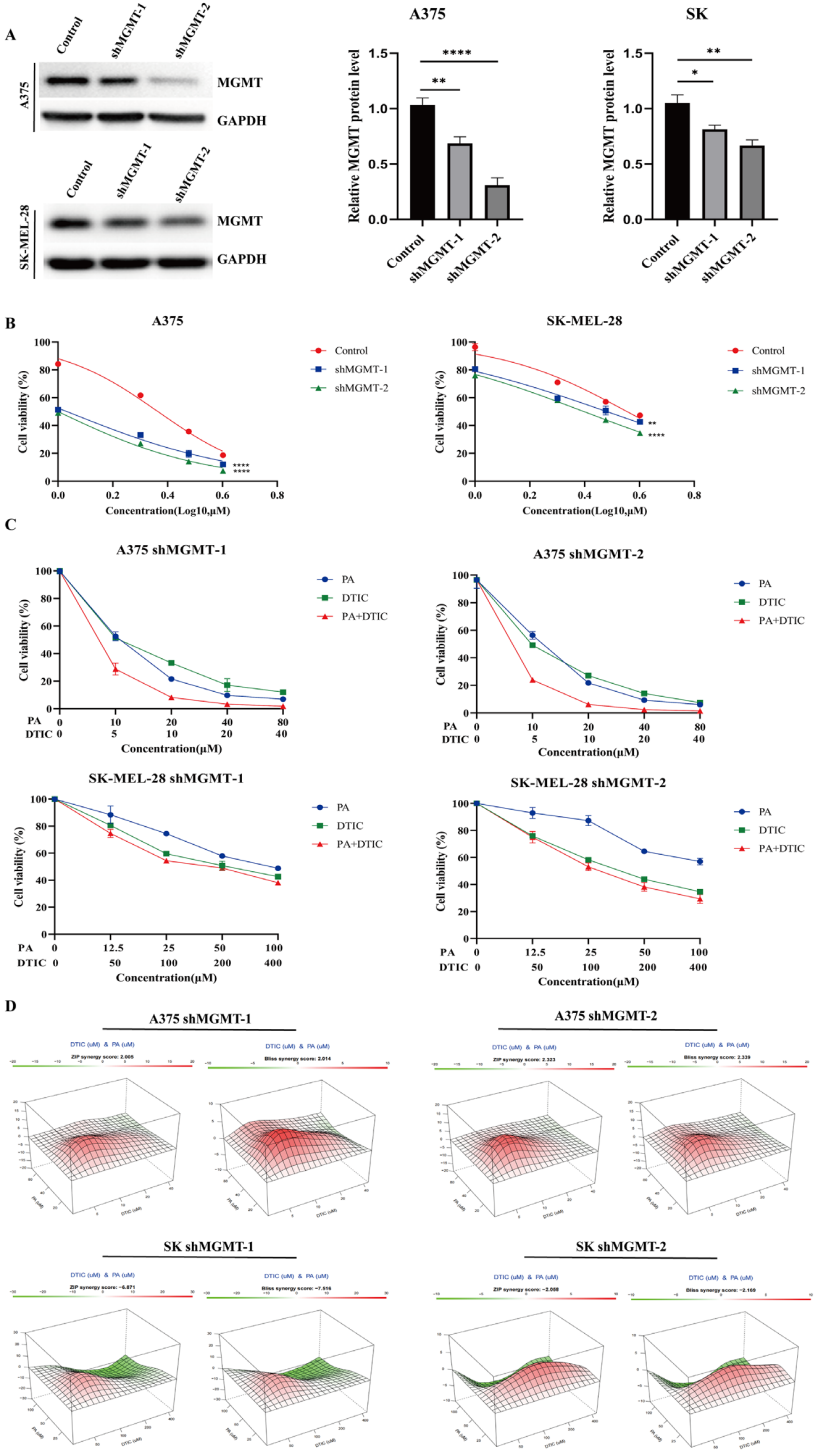
MGMT is required for PA-mediated synergistic effect. **A** The shRNA-mediated knockdown of MGMT was validated by western blot. Right panel represents the quantification of the MGMT protein levels in **A**. **B** Knockdown of MGMT increased the DTIC sensitivities in A375 and SK-MEL-28 cells. **C** Dose-response curves for MGMT-depleted melanoma cells treated with PA, DTIC or their combinations. The selected concentrations for treatment are identical with those in Fig. 1B. **D** Synergy scores were obtained from the data represented in **C**. The synergy scores were calculated by ZIP model and Bliss model, respectively. *p < 0.05, **p < 0.01 and **** *p *< 0.0001

To validate whether PA promoted cytotoxicity of DTIC via MGMT, we depleted MGMT expression in A375 and SK-MEL-28 by using two different shRNAs, and the shMGMT-1 showed slightly knockdown efficiency in comparison with the shMGMT-2 (Fig. 4A). With depletion of MGMT, A375 and SK-MEL-28 exhibited sensitivities to DTIC demonstrating that MGMT was involved in the DTIC resistance in the two melanoma cell lines (Fig. 4B). Further, we determined the synergy between PA and DTIC in the MGMT-depleted melanoma cells and found that knockdown of MGMT compromised the synergistic effects, suggesting that MGMT was required for the synergistic cytotoxicity of PA and DTIC (Fig. 4C-D).

## Discussion

In the past 10 years, immunotherapy and targeted therapy have largely improved the outcomes of melanoma patients. But with the emergence of drug resistance or unresponsiveness to immunotherapy, the DTIC/TMZ- based chemotherapy still has been placed as the first-line for advanced melanoma treatment. Because the low response rate and a few of patients have benefit from the regimen of the alkylating agent DTIC, the DTIC/TMZ- based chemotherapy was considered as palliative [23]. Recently, a retrospective analysis for patients with advanced melanoma from 24 melanoma centers reported that chemotherapy had a very limited effect on patients who failed to respond to immunotherapy [24]. Thus, exploration of novel chemotherapeutic agents to against advanced melanoma should be worthwhile.

Alkylating agents can generate 14 types of adducts, of which O6-MeG is one of the most potent adducts to generate DNA lesions and induce cell apoptosis [25, 26]. Repair of O6-MeG lesions is essential for cell genome integrity, and maintains cell survival. MGMT directly repairs O6-MeG by transferring the alkyl groups from the O6-MeG to the cysteine at the center of the catalytic pocket of the MGMT protein. Then, the O6-MeG covalently binds to the cysteine and inactivates MGMT, and the inactivated MGMT molecule undergoes ubiquitylation and degradation. This irreversible reaction consumes one MGMT molecule to remove one alkyl adduct [27–29]. Loss of MGMT expression allows O6-MeG to be more stable and persistent in a cell, which highlights the level of MGMT protein as a pivotal factor in O6-MeG repair [30, 31]. Transcriptional regulation of MGMT including promoter epigenetic modification ,has been extensively studied in cancers [32, 33]. Previously, PA has been implied as a transcriptional modulator through CtBP1 targeted [19]. In this study, we first examined the mRNA changes after PA treatment and found that the mRNA expression of MGMT was not affected by PA treatment, which was consistent with CtBP1 knockout in A375 and SK-MEL-28 (data not shown). The data suggested that PA-mediated transcription alteration was not involved in the regulation of MGMT in the two melanoma cell lines.

The proteasome and lysosome mediate two major ways for protein degradation, respectively [34]. It is well understood that MGMT is degraded by the ubiquitin-proteasome process [35, 36]. However, there is no clear evidence to support whether lysosome participates in MGMT degradation. In the present study, we found that MGMT protein was increased by DTIC treatment alone in A375 and SK-MEL-28 cells suggesting a positive cellular response to repair DTIC-induced genetic lesions. And PA reduced MGMT protein level by promoting its degradation in the two melanoma cell lines either by single usage or combination with DTIC. Mechanism study revealed that PA destabilized MGMT through the ubiquitin-proteasome process. However, further studies are required to reveal the detailed molecular mechanisms of MGMT ubiquitination triggered by PA.

As MGMT is critical for resistance to DTIC/TMZ, we depleted MGMT expression in A375 and SK-MEL-28 cells to test their synergies and sensitivities to DTIC. Both of the two cell lines compromised the synergistic effects and exhibited sensitivities to DTIC after knockdown of MGMT suggesting that MGMT expression is critical for PA-mediated synergy to DTIC. Additionally, with the depletion of MGMT, A375 acquired a more significant increase in sensitivity to DTIC than SK-MEL-28 did, implying that aside from MGMT, other factors were involved in DTIC resistance and may participate in the PA-mediated synergistic effects in melanoma cells. Because repair of DTIC-induced genetic lesions not only includes alkyl groups removal via MGMT but also involves other DNA damage repair pathways such as homologous recombination (HR) and non-homologous end joining (NHEJ), it is rational to focus on additional DNA repair proteins potentially regulated by PA [37, 38].

## Conclusion

Our study demonstrated that the bioactive compound, Protocatechuic aldehyde, synergistically promoted the cytotoxicity of DTIC to melanoma cells through the destabilization of MGMT protein. It could be a potential candidate for melanoma combinational chemotherapy.

## Electronic supplementary material

Below is the link to the electronic supplementary material.

**Additional file 1.**

## Data Availability

Data or materials will be made available on reasonable request to the corresponding authors, Yu Deng and Jian Li.

## Notes

The original online version of this article was revised: it was noted that due to a typesetting error the figure images were paired incorrectly and some information was lost.
