# Correction: Protocatechuic aldehyde acts synergistically with dacarbazine to augment DNA double-strand breaks and promote apoptosis in cutaneous melanoma cells

**DOI:** 10.1186/s12906-023-03965-2

**Published:** 2023-04-27

**Authors:** Junxia Pei, Zhou Su, Xin Zeng, Ya Zhong, Yamei Zhang, Yixi Yang, Qiuxia Lu, Jian Li, Yu Deng

**Affiliations:** 1 Engineering Research Center of Sichuan-Tibet Traditional Medicinal Plant, Chengdu University, Chengdu, 610106 China; 2 Institute of Cancer Biology and Drug Discovery, Chengdu University, Chengdu, 610106 China; 3 School of Food and Biological Engineering, Chengdu University, Chengdu, 610106 China; 4 School of Pharmacy, Chengdu University, Chengdu, 610106 China; 5 Key Laboratory of Clinical Genetics, Affiliated hospital of Chengdu University, Chengdu, 610106 China; 6 School of Basic Medical Sciences, Chengdu University, Chengdu, 610106 China

**Correction: BMC Complement Med Ther 23, 111 (2023)**

**https://doi.org/10.1186/s12906-023-03933-w**

Following the publication of the original article [1], it was noted that due to a typesetting error the figure images were paired incorrectly and some information was lost. The correct Figs. 1, 2, 3 and 4 are given below.

**Fig. 1:**
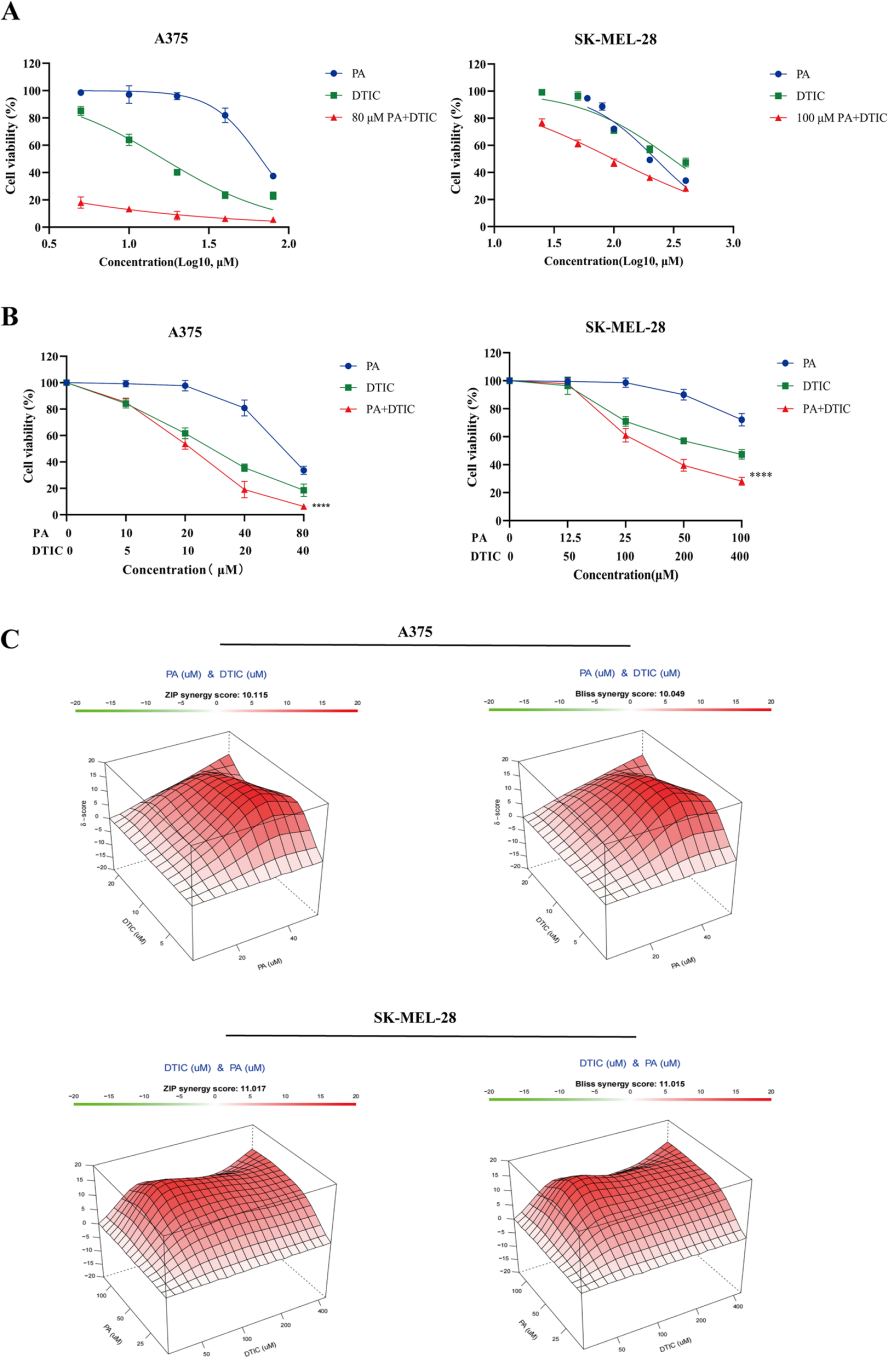
Protocatechuic aldehyde synergistically enhances DTIC cytotoxicity to melanoma cells. **A** Dose-response curves for PA, DTIC or DTIC combined with a certain concentration of PA for 72 h in A375 and SK-MEL-28 cells. **B** Dose-response curves for A375 and SK-MEL-28 cells treated with a range of concentrations of PA, DTIC or their combinations. **C** Synergy scores were calculated from the data represented in **B** for PA combined with DTIC for A375 and SK-MEL-28 cells. The left panel represents the synergy scores from ZIP model. The right panel represents the synergy scores from Bliss model. **** *p*<0.0001

**Fig. 2:**
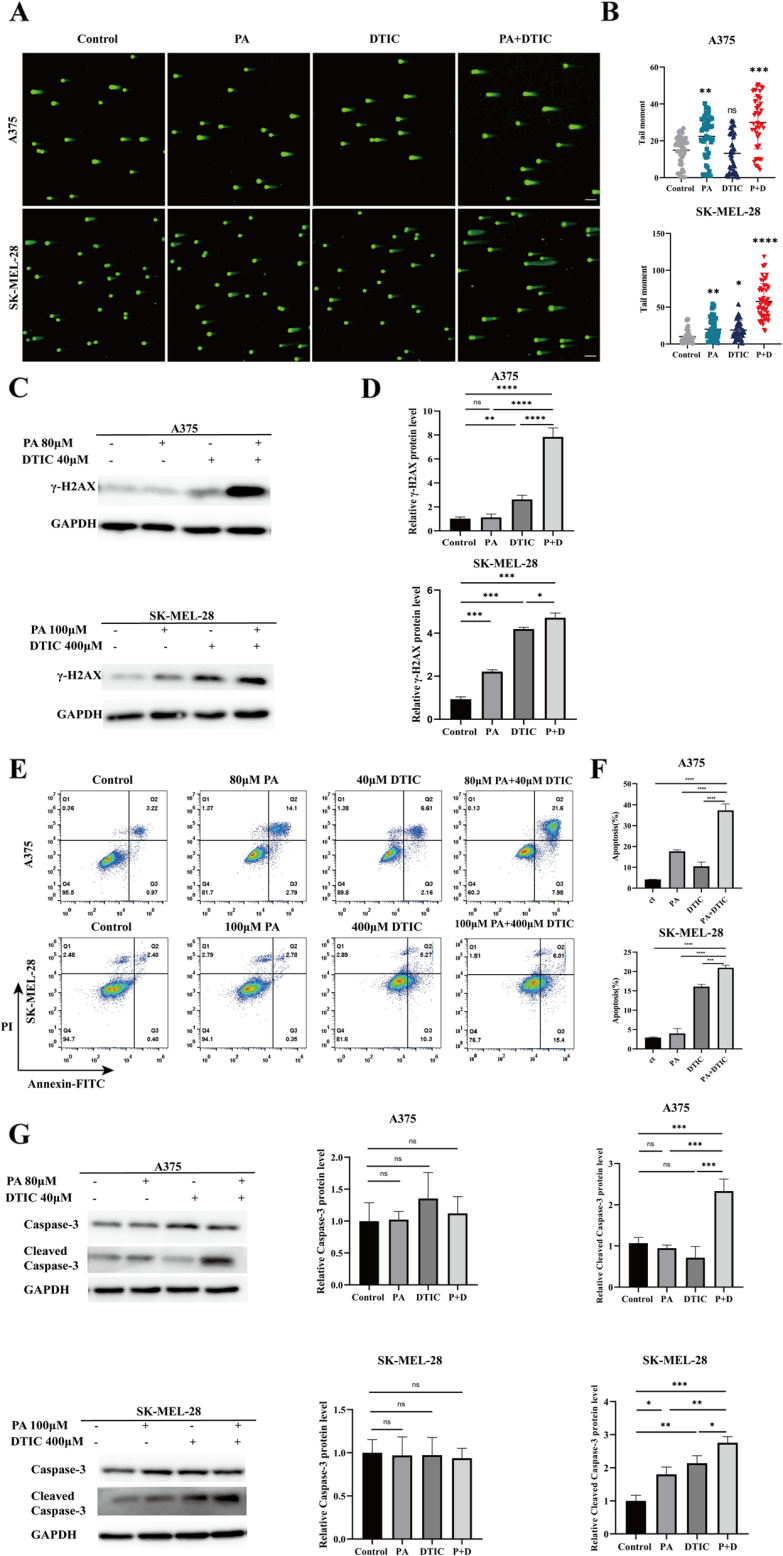
Combination of PA and DTIC increased DNA-double strand breaks and apoptosis in A375 and SK-MEL-28 cells. **A** The double-strand breaks in A375 and SK-MEL-28 cells were assessed by neutral comet assay. **B** Quantification of DNA percentages in comet tails in A. **C** γ-H2AX levels after 72 h of treatment with DMSO, PA, DTIC or combination of PA and DTIC in A375 and SK-MEL-28 cells. **D** Quantification of the relative γ-H2AX levels in **C**. **E** Cell apoptosis was analyzed by flow cytometry. The combined treatment showed the most apoptosis induction. **F** Quantification of apoptosis ratios in **E**. **G** Cleaved caspase-3 protein levels after 72 h of treatment with DMSO, PA, DTIC or combination of PA and DTIC in A375 and SK-MEL-28 cells. Right panel represents quantification of the relative cleaved caspase-3 protein levels in **G**. ns = no significant,*p < 0.05, **p < 0.01,***p < 0.001 and **** *p*<0.0001

**Fig. 3:**
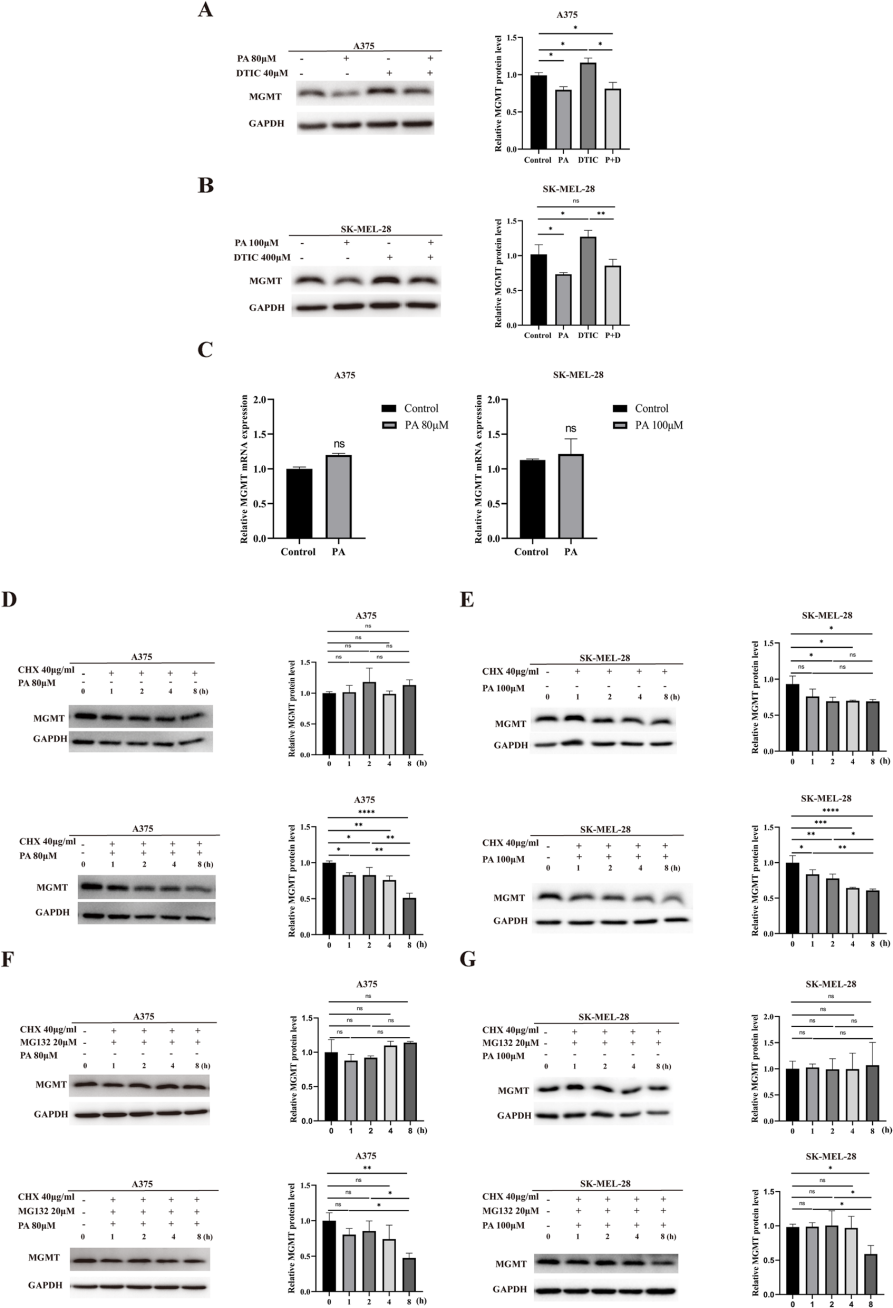
PA promotes MGMT degradation in melanoma cells. **A-B** Left panel shows western blot analysis for MGMT levels in A375 and SK-MEL-28 cells after 72h of treatment with DMSO, PA, DTIC or combination of PA and DTIC. Right panel represents the quantification of the MGMT protein levels for the left panel. **C** MGMT mRNA expression in A375 and SK-MEL-28 after 72 h PA treatment. **D-E** A375 and SK-MEL-28 were treated with cycloheximide in the presence or absence PA for 0-8 h hours. Western blot showed the MGMT degradation rates. Right panel represents quantification of the MGMT protein levels. **F-G** A375 and SK-MEL-28 were treated with MG132 combined with cycloheximide in the presence or absence of PA for 0-8 h hours. The MGMT protein levels were analyzed by western blot. Right panel represents quantification of the MGMT protein levels. ns = no significant, *p < 0.05, **p < 0.01,***p < 0.001 and **** *p*<0.0001

**Fig. 4:**
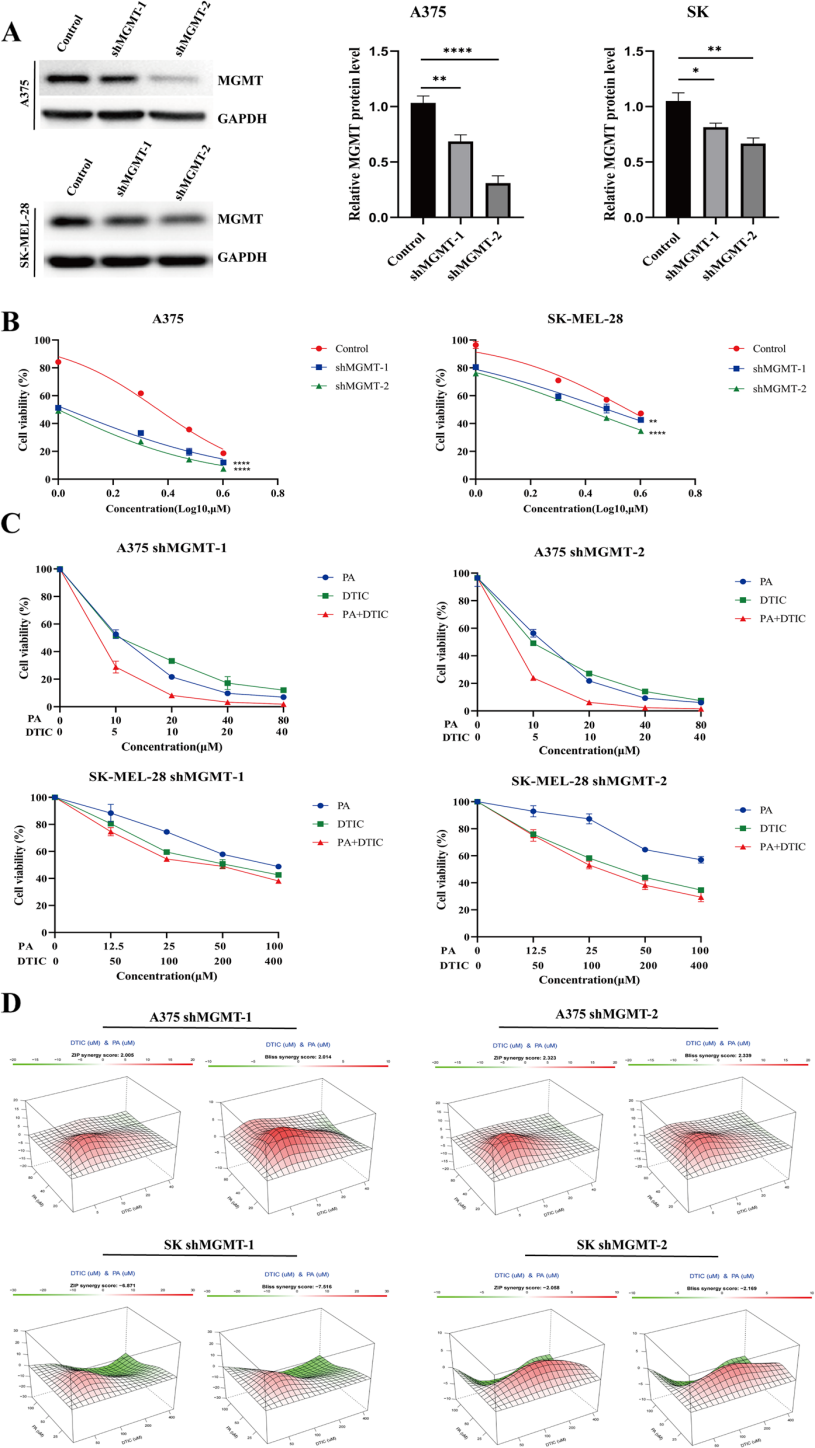
MGMT is required for PA-mediated synergistic effect. **A** The shRNA-mediated knockdown of MGMT was validated by western blot. Right panel represents the quantification of the MGMT protein levels in **A**. **B** Knockdown of MGMT increased the DTIC sensitivities in A375 and SK-MEL-28 cells. **C** Dose-response curves for MGMT-depleted melanoma cells treated with PA, DTIC or their combinations. The selected concentrations for treatment are identical with those in Fig. 1B. **D** Synergy scores were obtained from the data represented in **C**. The synergy scores were calculated by ZIP model and Bliss model, respectively. *p < 0.05, **p < 0.01 and **** *p* < 0.0001

The correct figures and captions have been included in this correction, and the original article has been corrected.
