# Supplementary material for: Protocatechuic aldehyde acts synergistically with dacarbazine to augment DNA double-strand breaks and promote apoptosis in cutaneous melanoma cells
Source: BMC Complement Med Ther. 2023 Apr 6;23:111. doi: 10.1186/s12906-023-03933-w (PMC10077623; doi:10.1186/s12906-023-03933-w)

Figure 2C

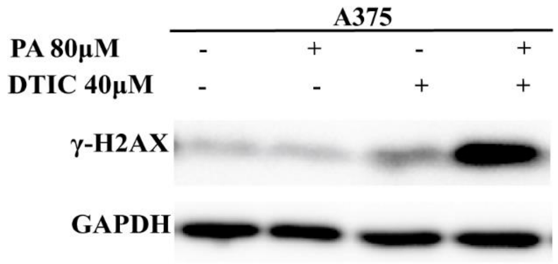

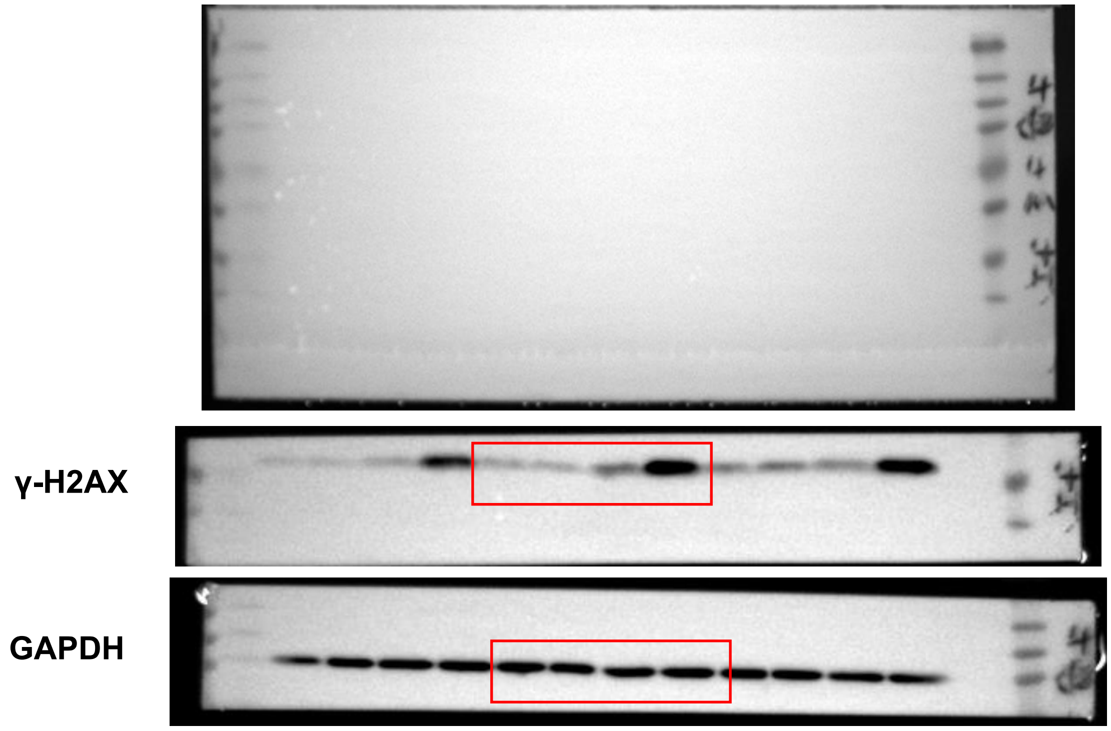

Figure 2C

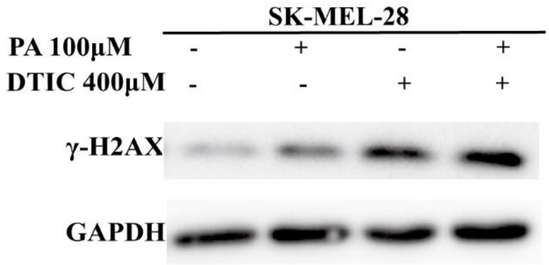

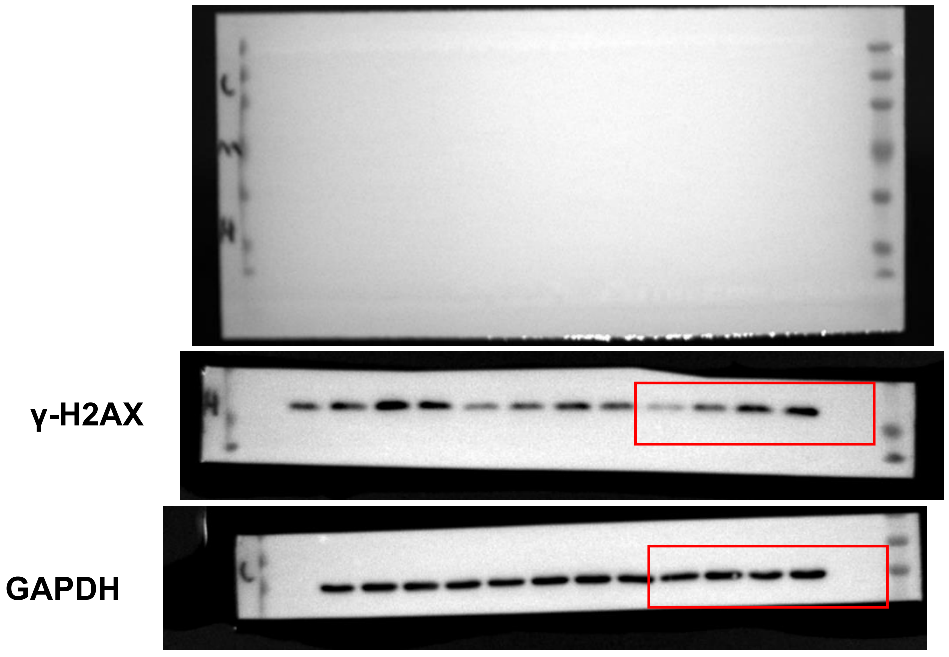

Figure 2G

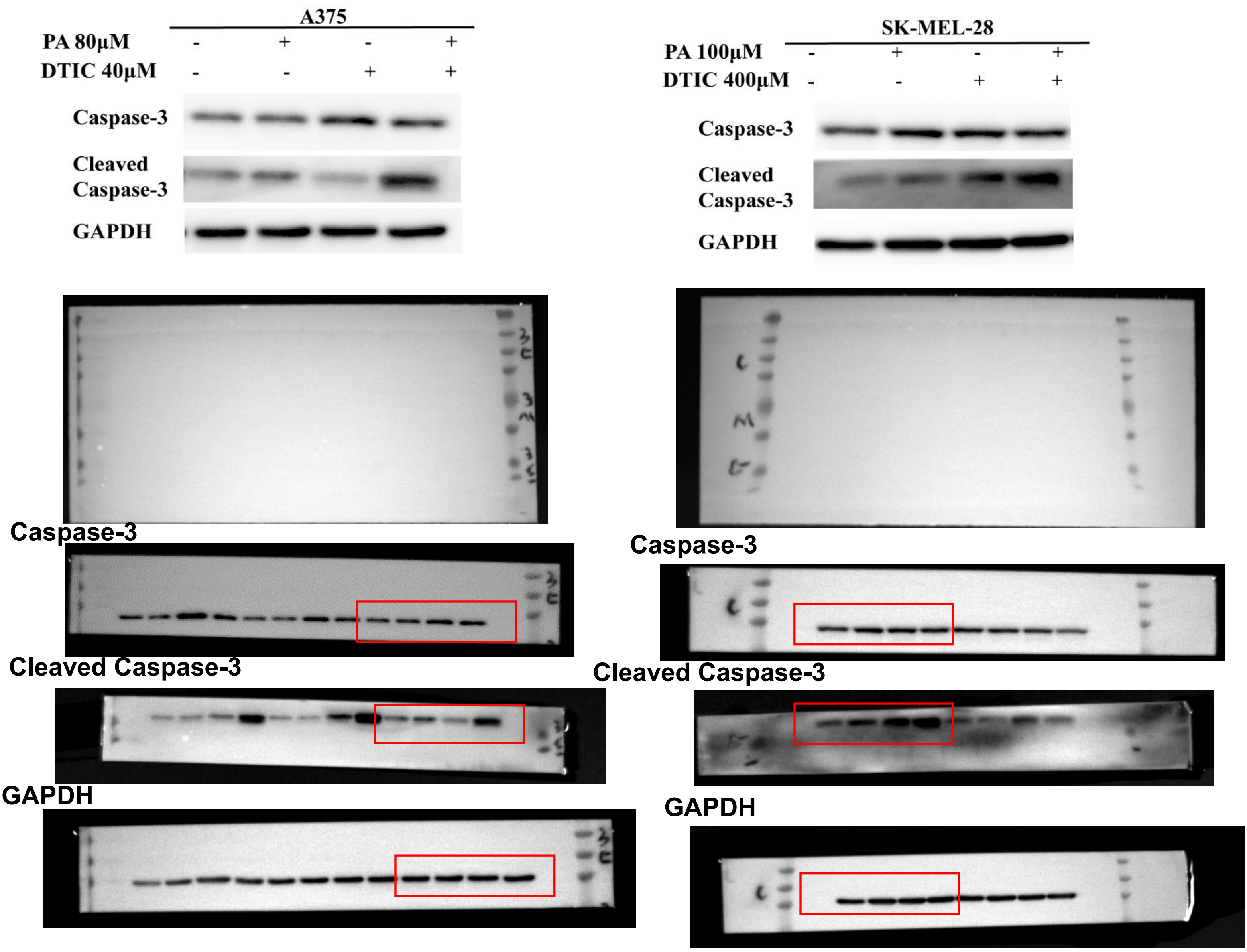

Figure 3A

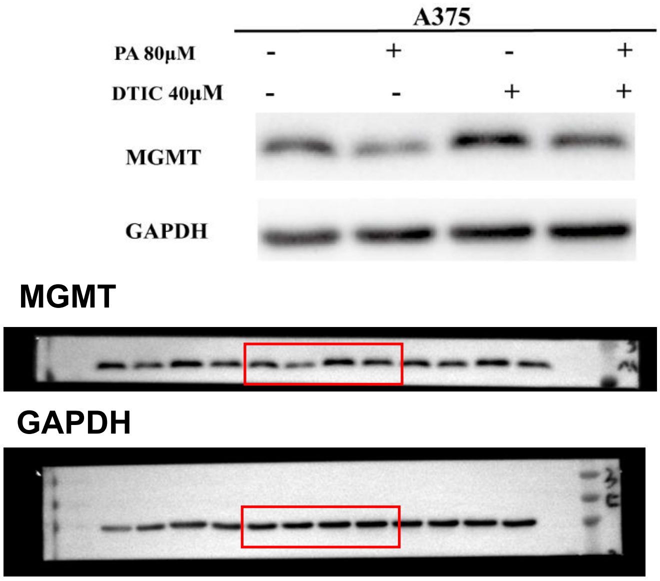

Figure 3B

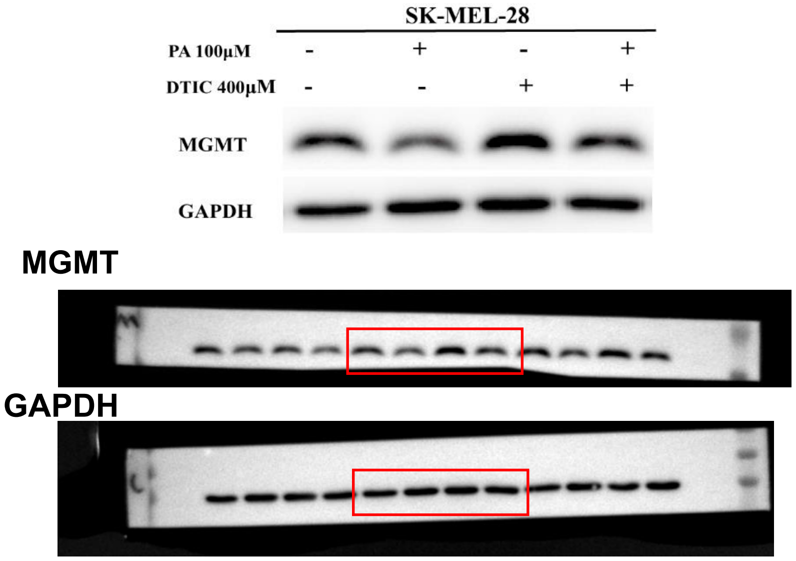

Figure 3D

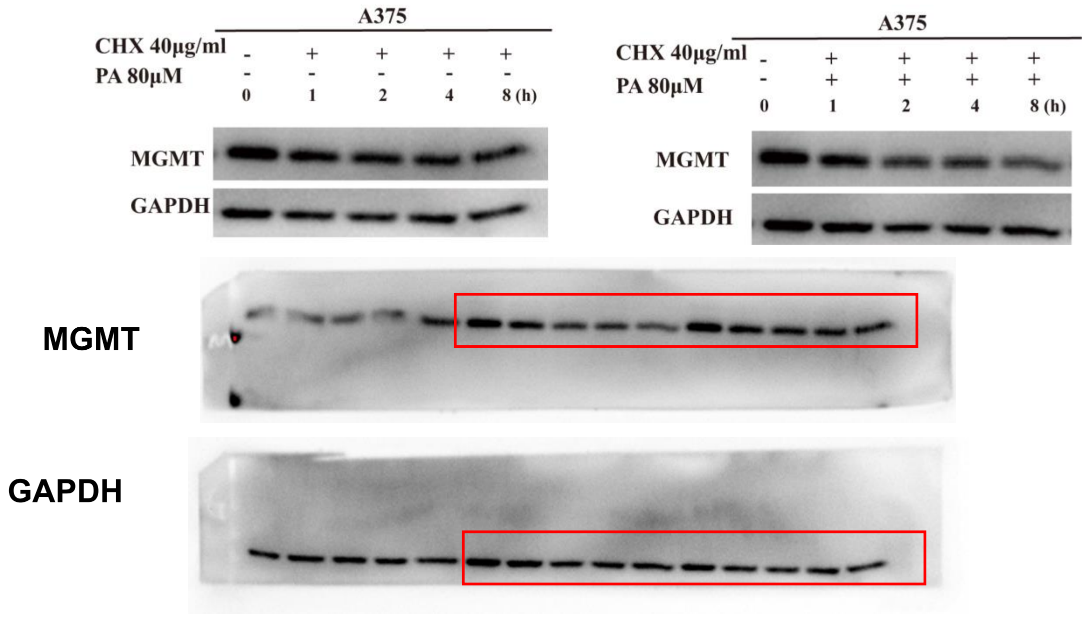

Figure 3E

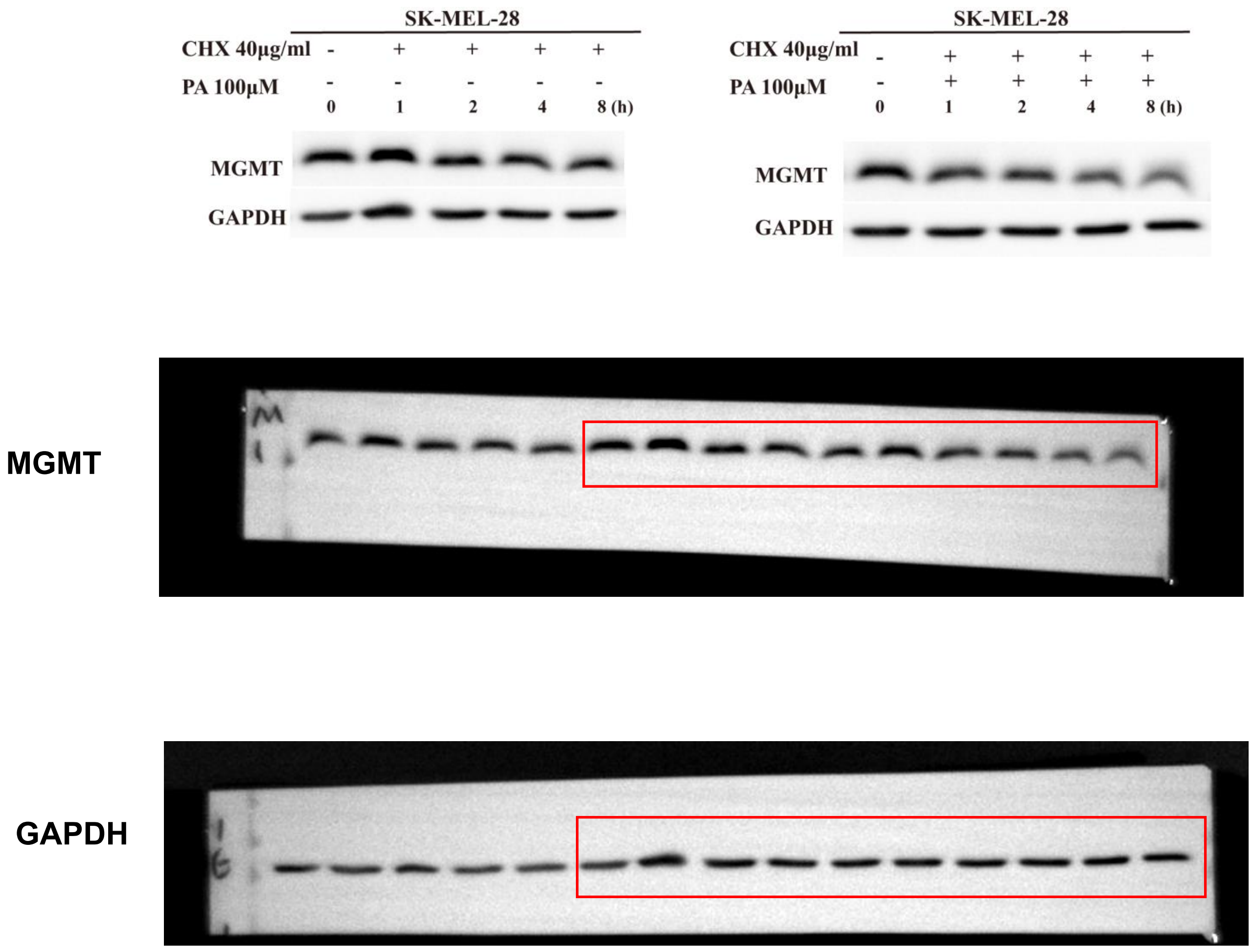

Figure 3F

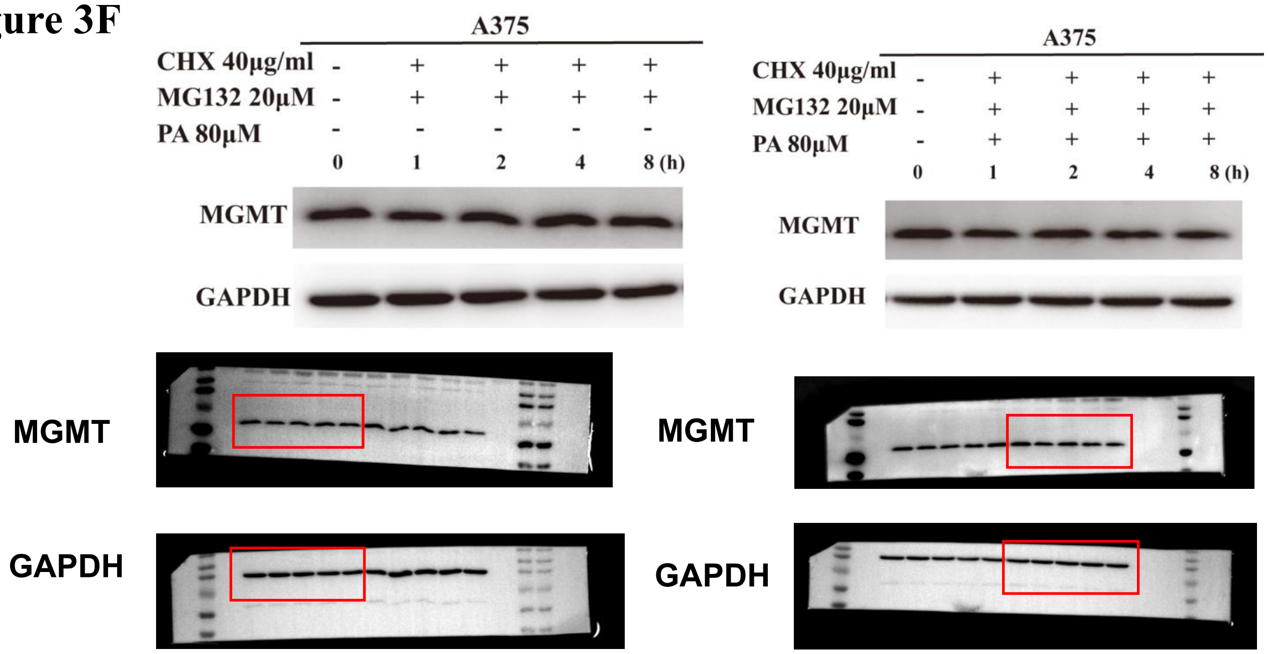

Figure 3G

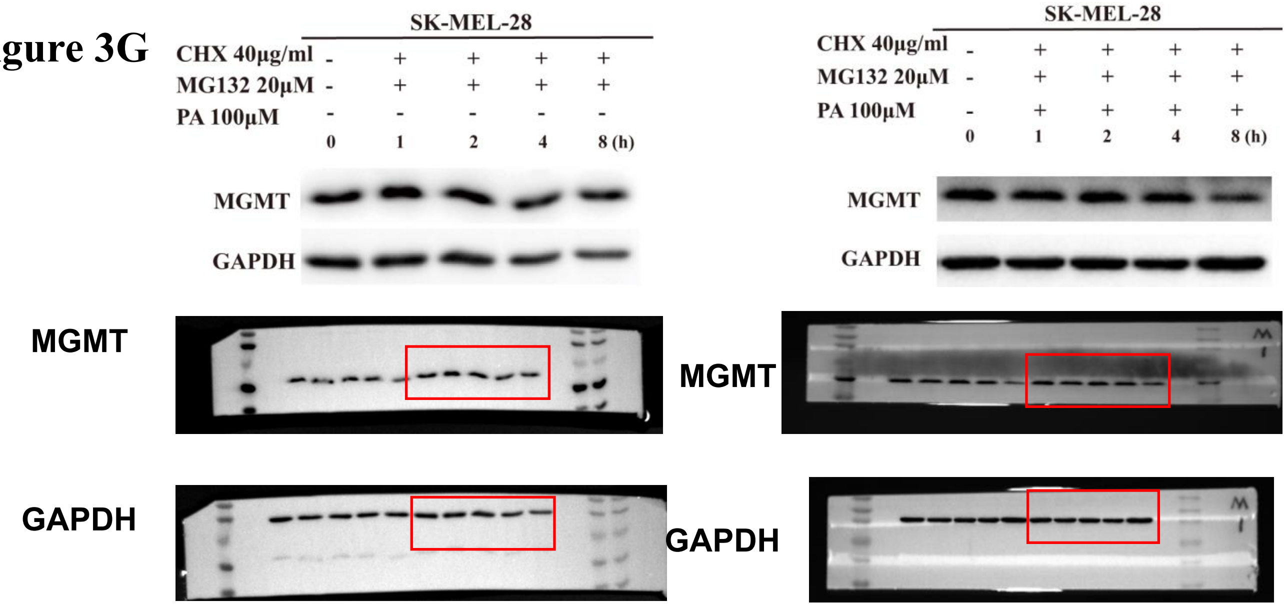

Figure 4A

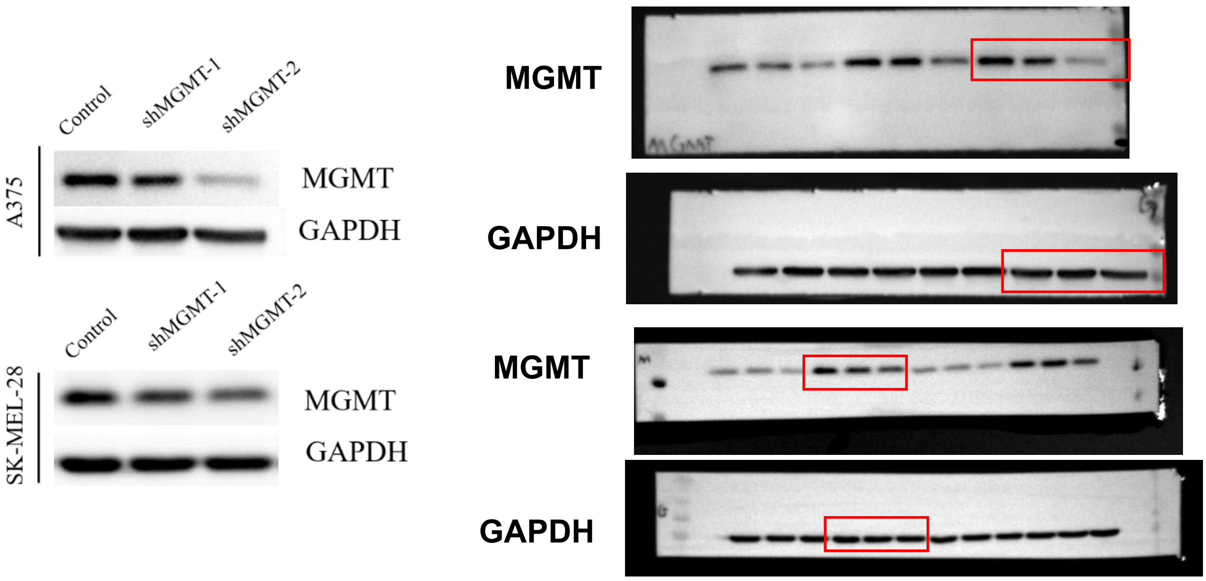

Supplement: Supplementary file 1 — Additional file 1. [file 12906_2023_3933_MOESM1_ESM.pdf]
